# Physicochemical Characteristics of Meat Analogs Supplemented with Vegetable Oils

**DOI:** 10.3390/foods12020312

**Published:** 2023-01-09

**Authors:** Youngjae Cho, Junhwan Bae, Mi-Jung Choi

**Affiliations:** 1 Department of Food Science and Technology, Pusan National University, 1268-50 Samrangjin-ro, Miryang 50463, Republic of Korea; 2 Department of Food Science and Biotechnology of Animal Resources, Konkuk University, 120 Neungdong-ro, Gwangjin-gu, Seoul 05029, Republic of Korea

**Keywords:** meat analog, vegetable oil, vegetable protein, storage stability, oil content, antioxidants, limonene, orange oil

## Abstract

This study identified the effect of the type and concentration of vegetable oil on the quality of meat analogs and analyzed the differences in their physiochemical characteristics. Various vegetable oils, such as castor oil, orange oil, palm oil, shortening, and margarine, were added to meat analogs. The meat analog was prepared by adding 10, 20, 30, 40, and 50 g of each vegetable oil based on 100 g of textured vegetable protein. The cooking loss, water content, liquid-holding capacity, texture, and antioxidant content of the meat analogs were assessed, and a sensory evaluation was performed. The meat analog with orange oil had a higher water content than the others, regardless of the amount of added oil, and it had a relatively high liquid-holding capacity. The DPPH(2,2-diphenyl-1-picrylhydrazyl) radical scavenging activity of the meat analog with orange oil was higher than that of the others. The sensory evaluation also showed a decrease in soy odor and an increase in juiciness. Therefore, adding orange oil improves the preference, juiciness, soy odor, and quality of meat analogs. Our results demonstrate that orange oil has positive effects on the productivity of meat analogs and can help to improve meat analog consumption.

## 1. Introduction

Worldwide meat production, including beef, pork, chicken, and lamb, is approximately 19 billion kg per year, and per capita meat consumption is expected to increase from 8.2 kg in 2017 to 8.6 kg in 2025 [1,2], possibly due to higher incomes and increased stability of life [3]. However, while demand increases, meat supply is limited because the resources to raise livestock, such as land and water, are finite [4,5]. Additionally, greenhouse gases emitted from livestock cause environmental problems. Methane emissions are approximately 37% and nitrous oxide is approximately 65% of global emissions [6]. carbon dioxide emissions also continue to increase [7]. In terms of health, meat is high in cholesterol and saturated fat, and contributes to high blood pressure, obesity, cardiovascular disease, and cancer [8,9,10].

As meat consumption has increased, the demand for meat substitutes has also increased; interest in developing those based on plant protein is growing. Soybean protein-based meat substitutes have been studied [11,12]; however, they still lack a substitute for meat gravy, and are easily broken and difficult to chew, and therefore, they do not satisfy consumers’ preferences [13]. In addition, it is hard to remove the odor from soy protein-based products; therefore, research is needed to mask it [14]. The degree of odor was compared with that of powder prepared from raw soybean via heat treatment and then added to soybean meat [15]. Soybean odor was reduced by treating cutlet-type soybean instead of pork with Protamex enzyme [16]. In addition, the effect of adding carrageenan on the succulence of meat substitutes was reported [17]. Most studies report improved texture with chewiness similar to meat [11,18]. However, further research is needed to improve the succulence and soybean odor.

The fundamental purpose of alternative meat development is to imitate the taste and flavor using plant materials, while preventing problems from excessive meat consumption, and satisfying consumers’ desire for meat. The characteristics of the oils used in meat analog are as follows: vegetable shortening and margarine, with a relatively high saturated fat content, are the most similar to animal oil in composition because they artificially form saturated fats while undergoing hydrogenation [19]. In addition, orange oil, which might attenuate the bean odor of meat substitutes, was selected as a flavor ingredient with high unsaturation and volatility [20]. Ahmad [21] reported that the odor was attenuated using the flavor component of orange oil. Castor oil is known to have a high viscosity among vegetable oils [22], and it was selected for possible high retention in substitute meat. Although palm oil has not undergone hydrogenation as shortening or margarine have, it exists as a solid at room temperature owing to its relatively low unsaturation [23] and was selected for the potential to improve retention and succulence.

The purpose of this study was to improve the succulence and odor of plant meat. Various vegetable oils were added at different concentrations; the characteristics of the samples were compared and the quality change according to storage temperature and period was confirmed.

## 2. Materials and Methods

### 2.1. Materials

Textured vegetable protein (TVP, Supromax 5050^®^, Supromax 5010^®^, Solae do Brasil Ind. e Com. A), soy protein isolate (SPI, Avention, Incheon, Korea), and binder (Meatline^®^ 2714, Danisco, Copenhagen, Denmark) were combined. The meat analog was prepared by adding castor oil (Daejung Chemicals, Siheung, Gyeonggi, Korea), orange oil (Sigma-Aldrich Inc., St. Louis, MO, USA), palm oil (Lottefoods, Cheonan, Korea), margarine (Ottogi, Anyang, Korea), or shortening (Hain Celestial Group, Inc., Lake Success, NY, USA) to the mixture.

### 2.2. Preparation of Vegetable Protein

Meat analog was prepared by partially modifying the manufacturing process provided by Daesang Co., Ltd. TVP Supromax 5050^®^ and Supromax 5010^®^ were hydrated in distilled water 10 times their weight for 2 h and dehydrated for 5 min using a dehydrator (WS-6600, Hanil Electric, Seoul, Korea). The dehydrated TVP Supromax 5050^®^ and Supromax 5010^®^ in a 1:2 ratio, SPI, binder, and five types of vegetable oil were placed in a hand blender (Multiquick 3 Vario MQ 3145, Braun, Kronberg im Taunus, Germany) according to the composition ratio in Table 1 and mixed for 90 s. After placing 19 g of the mixed dough into a stainless-steel cylindrical mold and forming it, it was placed in an oven (M4207, Simfer, Istanbul, Turkey), preheated to 180 °C, and baked for 7 min, turned over, and baked again for 7 min. The cooked meat analog was left to cool to room temperature for 30 min and then used in the experiment.

### 2.3. Experimental Method

#### 2.3.1. Cooking Loss

Cooking loss was calculated by substituting the weight of meat analog before and after cooking using the formula below. After cooking, the weight was measured, and the samples were left to cool at room temperature for 30 min.

Cooking loss % = {(W_1_ − W_2_)/W_1_} × 100

W_1_: Weight of sample before cooking (g)

W_2_: Weight of sample after cooking (g)

#### 2.3.2. Water Contents

The outer part of the cooked meat analog was removed, and the inner part was used. The water content of 1 g of each meat analog dough and cooked meat analog was measured by atmospheric pressure heating and drying according to the AOAC method [24].

water contents % = {(W_1_ − W_2_)/W_1_} × 100

W_1_: Weight of sample before drying (g)

W_2_: Weight of sample after drying (g)

#### 2.3.3. Liquid-Holding Capacity

The liquid-holding capacity (LHC) of the dough and the cooked meat analog was measured simultaneously with the water and oil retention by modifying the method of Wierbicki and Deatherage [25]. Each sample was placed in a 15 mL conical tube containing 1 g of sterile gauze and stored overnight at 4, 25, and 35 °C; centrifugation was then performed for 10 min at 3000 rpm (Labogene 1736R, GYROZEN Co., Ltd., Kimpo, Korea). The weight of the sample before and after centrifugation was substituted into the formula below and the value was calculated.

Liquid-holding capacity % = {(W_1_ − W_2_)/W_1_} × 100

W_1_: Sample weight before centrifugation (g)

W_2_: Sample weight after centrifugation (g)

#### 2.3.4. Texture Measurement

The texture of the meat analog was measured using a texture analyzer (CT3-1000; Brookfield Engineering Laboratories, Inc., Middleboro, MA, USA) by cutting the sample into cubes with a width, length, and height of 2 cm after standing to cool. The measurement was performed under the conditions of a strain rate of 40%, measurement speed of 2.5 mm/s, and trigger load of 10 g. Measurements were repeated 10 times per treatment group using a cylindrical probe (TA4/1000).

#### 2.3.5. DPPH Radical Scavenging Activity

DPPH (2,2-diphenyl-1-picrylhydrazyl) (Sigma-Aldrich, St. Louis, MO, USA) free-radical scavenging activity of cooked meat analog was measured by modifying the method described by Blois [26]. One gram of freeze-dried meat analog and 25 mL of 70% ethanol was subjected to hot water extraction for 3 h in a water bath (BF-30SB; Biofree, Seoul, Korea) set at 80 °C. The extract was filtered using filter paper (Whatman No. 2, Healthcare Life Science, Buckinghamshire, UK) and then concentrated using a reduced pressure concentrator (EYELA rotary evaporator N-1000, SUNILEYELA, Seongnam, Korea). The concentrate was freeze-dried (MCFD8512, Ilshinbiobase Co., Dongducheon, Korea) to obtain a powder, which was prepared at a concentration of 1 mg/mL in distilled water and used as the sample. After reacting 0.1 mL of sample with 0.1 mL of 0.2 mM DPPH reagent in the dark at room temperature for 30 min, absorbance was measured at 517 nm using a spectrophotometer (Multiskan GO, Thermo Scientific, Waltham, MA, USA). As a control, to which the sample was not added, 0.1 mL of methanol was used, and the measurement was performed in the same manner. To correct the absorbance value for the color of the sample itself, methanol was added instead of the DPPH reagent, and the absorbance was measured in the same way. The value was calculated by substituting the measured absorbance with the following formula:

DPPH radical scavenging activity % = [1 − {(A1 − A2)/A3}] × 100

A1: Absorbance of 0.1 mL DPPH solution + 0.1 mL sample solution

A2: Absorbance of 0.1 mL methanol + 0.1 mL sample solution

A3: Absorbance of 0.1 mL DPPH + 0.1 mL methanol

#### 2.3.6. Sensory Test

The sensory characteristics of meat analog supplemented with the five types of vegetable oils were determined at a concentration of 30 g/100 g TVP. The concentration of oil added to the sample used for sensory testing was selected to be close to the fat content of Beyond Meat hamburger patty products. Prior to sensory testing, the Institutional Review Board (IRB) of Konkuk University approved the sensory test in accordance with the Guidelines for Good Clinical Practice by the International Conference on Harmonization (ICH GCP) to protect the reliability of the experiment and the human rights of the evaluators (700355- 201901-HR-294). Ten sensory panelists who had received regular training for 4 months, identified and evaluated the sensory characteristics of the plant meat. Samples were cut to the same size and a 3-digit random number was assigned to each sample. Hardness, succulence, soybean odor, strength, preference for oily taste, and overall preference were evaluated. An 11-point scale was used, and the higher the score, the higher the intensity and preference.

#### 2.3.7. Statistical Analysis

The analysis in this study was conducted more than three times. Physicochemical analysis results were analyzed using SPSS statistical program (Statistical Package for the Social Science, Ver. 24.0 IBM., Chicago, IL, USA). One-way ANOVA was used to determine the differences between samples, and significant differences were verified using Duncan’s multiple range test for post hoc testing. An independent samples t-test was used to confirm the differences depending on whether cooking was performed.

## 3. Results and Discussion

### 3.1. Cooking Loss

In this experiment, the weight of meat analog produced by adding five different types of vegetable oils at various concentrations was measured before and after cooking; the weight loss after cooking is shown in Table 2. In the sample containing orange oil, the weight loss after cooking increased as the concentration increased (*p* < 0.05), whereas it decreased when castor oil was added. When palm oil, shortening, and margarine were added, the cooking loss decreased as the concentration increased, but increased in the P50, S30, and M30 samples. In addition, when the same concentration of oil was added, except for a concentration of 10 g/100 g TVP, the sample with no elution had a lower cooking loss than the sample with orange oil added (*p* < 0.05). Among the samples from which the oil was eluted, the cooking loss of the sample with added shortening was significantly higher (*p* < 0.05). With palm oil, the cooking loss increased by 10.33% while the oil eluted at P50; with shortening and margarine, oil elution started to occur from S30 and M30, and in S50 and M50, the cooking loss increased by 10.61% and 10.21% compared to before elution, respectively. The reason for the decrease in cooking loss is that more moisture is lost than oil during the cooking process [27]. When the amount of the sample was the same, the concentration of the added oil increased, possibly because the amount of water present in the sample decreases as it increases. In addition, cooking loss decreases and then increases at a certain oil concentration; when even more oil is added, it is visibly eluted during cooking. Therefore, the weight of the sample was greatly reduced after cooking. On the other hand, because of the relatively high water solubility of limonene, the sample with added orange oil had a content of 92–95% of the total components of orange oil [28]; when water is lost during the cooking process, it seems that the loss on heating is increased because the total weight is greatly reduced compared to other oils as the limonene component is lost.

### 3.2. Water Contents

Table 3 shows the water content of the meat analog dough and cooked meat analog prepared by adding five types of vegetable oils at each concentration. Except for orange oil samples, the water content decreased significantly as the concentration increased, whether it was cooked or not (*p* < 0.05). In the orange oil samples, the water content increased as the oil concentration increased (*p* < 0.05), and it always had a significantly higher water content than the other samples whether cooked or not, and regardless of the concentration (*p* < 0.05). The water content of the dough and cooked samples of O10 were 61.61% and 63.34%, respectively, which were 4.09% and 5.38% higher than those of C10, respectively, which had the lowest water content at the same concentration. As the oil concentration increased, the difference in water content between the orange oil sample and the other samples became larger. In addition, the O50 was 66.29% for the dough and 67.26% for the cooked samples, showing 22.26% and 24.57% higher water content than C50, respectively. On the other hand, Zhang et al. [29] found that the higher the sausage fat content, the lower the water content. In general, when samples are prepared with the same weight, the amount of water decreases as the amount of oil increases. In this experiment, the water content of the samples, except the orange oil sample, showed the same phenomenon; the orange oil sample showed the opposite result due to the relatively high water solubility of limonene; this accounts for 92–95% of orange oil, as mentioned in the heat loss results [28]. It seems that the difference in sample weight before and after drying is large compared to other samples because limonene components are lost in addition to water.

Among the samples showing a significant difference in water content before and after cooking, the content increased after drying, except for P20, P50, and C50. It seems that the loss of components other than water during the cooking process is greater than that of water; therefore, the water content of the sample is relatively high. In a study by Kim et al. [30], if the weight loss of the entire sample is greater than the loss of specific components during the heat treatment process, the content of specific components may be relatively increased after heat treatment; however, it is difficult to explain the increase due to the production of the component.

### 3.3. Liquid-Holding Capacity

Liquid-holding capacity was measured after storing the meat analog dough and cooked meat analog at 4, 25, and 35 °C overnight at each concentration of five types of vegetable oils (Table 4, Table 5 and Table 6). Castor oil and orange oil have melting points below 0 °C, but palm oil, shortening, and margarine exist as a solid at 4 °C and coexist as a solid and a liquid at 25 °C. In addition, because it exists in a liquid state at 35 °C, samples were stored at different temperatures in order to determine the difference in liquid-holding power according to the state of oil. The samples were stored overnight at each temperature to ensure a sufficient phase change of the oil, and the liquid retention was measured. The liquid-holding capacity showed a tendency to decrease as the storage temperature increased, and the decrease was greater in the samples containing palm oil, shortening, and margarine (*p* < 0.05). Cooked S50 showed a liquid retention of 95.89% when stored at 4 °C, which decreased by 18.77% at 35 °C to 77.12%, and M50 dough decreased by 18.37% from 93.60% to 75.25%. This is due to the difference in the bonding force between oil molecules according to the state of the oil at each temperature. That is, the higher the bonding force between the oil molecules in the sample, the lower the amount eluted to the outside when a physical force is applied [31]. In addition, in general, the closer the liquid is to the solid state, the higher the bonding force between the oil molecules; therefore, the liquid-holding power is higher [32].

In contrast, water has a stronger binding force with proteins than oil because of hydrogen bonding [33]. In this experiment, because the samples were the same weight, if the volume of oil increased, the proportion of hydrated soy protein decreased; thus, the amount of water present decreased, possibly due to the volume of water strongly binding to soybean protein decreasing [29].

When the plant meat was stored at 4 °C, the liquid retention showed a tendency to increase, except for S10 and S30, among samples that showed a significant difference after cooking, and C30 showed a 5.83% increase in liquid retention after cooking. Additionally, when stored at 25 °C and 35 °C, the liquid-retention capacity increased as in the sample stored at 4 °C, except for some of the margarine and shortening samples, among the samples with the difference. In addition, C50 increased liquid-holding capacity by 7.94% and 10.08% at 25 °C and 35 °C, respectively. The experimental results were similar to that of Kim et al. [34], in that water absorption increased after cooking soybean protein. During the cooking process, as the protein structure is denatured by heat, active groups such as OH, SH, COOH, and NH_2_ groups that were not originally observed appear on the surface, increasing the reactivity. As a result, the water absorption-capacity of soybean protein increases, and the longer the heating time, the greater the water absorption capacity of soybeans [35,36]. In this experiment, there was also a tendency for the liquid-retention capacity to increase after cooking, possibly due to the increase in the active group of the denatured soybean protein and the binding force with water due to hydrogen bonding.

Because palm oil, shortening, and margarine have melting points higher than 4 °C, when measuring the liquid retention of a sample stored at 4 °C, the oil within exists as a solid. It was determined that the bonding force between the oil molecules and the liquid-holding power were both high. The orange oil samples have a relatively high liquid retention capacity under all conditions because of the hydrogen bonding between water-soluble limonene and soy protein [28,36]. Castor oil is known to have very high viscosity, unlike other vegetable oils [37], but it is not considered to have a significant effect on liquid retention.

### 3.4. Texture and pH Measurement

Table 7 and Table 8 show the texture of cooked meat analog with five kinds of vegetable oils added at different concentrations. In addition to adhesiveness, there were differences in hardness, cohesiveness, springiness, gumminess, and chewiness. Hardness is essential to the suitability of food texture and is an important quality constraint [38]. In this experiment, the hardness decreased as the amount of oil increased; the hardness of the samples with orange oil were significantly higher than the others (*p* < 0.05). When the amount of oil added was the same, O10 had a hardness of 12.09 N (1234 g) higher than that of M10, and O50 had a hardness of 10.64 N (1086 g) higher than that of S50. These results can be explained in relation to the pH values of the vegetable oil, as shown in Table 9. The pH of the orange oil (3.19 ± 0.04) was closest to pH 4, near the isoelectric point of soy protein; therefore, denaturation during cooking occurred the most in the samples with orange oil [39]. It may be because a rigid matrix structure is formed by the hydrophobic interactions between soy proteins [40]. In contrast, the samples with margarine and shortening showed pH values of 6.31 ± 0.04 and 6.51 ± 0.05, respectively, a relatively large difference from pH 4. Accordingly, the protein matrix structure is loose compared to the samples with other oils, showing relatively low hardness. The decrease in hardness with an increase in the amount of oil added is due to the protein density of the sample decreasing when the proportion of hydrated soybean protein decreases [41]. In contrast, stickiness and chewiness refer to the force and energy required to break down food until it can be swallowed [42].

Springiness refers to the property of returning to its original state after the force applied to the sample is removed, and cohesiveness is an indicator of the strength of the internal bonds of food. Therefore, it is possible to measure tissue-forming ability using chewing [42,43]. In this experiment, although there was a significant difference between the samples, owing to small deviations in elasticity and cohesiveness, no clear trend was observed.

### 3.5. DPPH Radical Scavenging Activity

DPPH is a compound with relatively stable radicals and is widely used to confirm the antioxidant ability by reducing antioxidants [44], using the degree of purple decolorization as an index of reduction by aromatic compounds and amines due to the electron-donating ability of antioxidants [45]. The DPPH radical scavenging activity of the meat analog samples was analyzed, and the results are shown in Table 10. Scavenging activity increased as the concentration of vegetable oil increased, and that of the sample with orange oil was significantly higher at all concentrations (*p* < 0.05). The scavenging activities of O10 and O50 were 30.15% and 36.91%, respectively, and all samples containing orange oil exhibited more than 30% scavenging activity. The remaining samples showing more than 30% scavenging activity were P30, P40, and P50, with scavenging activities of 30.19%, 31.46%, and 33.46%, respectively. In addition, the sample containing orange oil showed more than 7.79% higher scavenging activity than the sample with the lowest scavenging activity at each concentration; the scavenging activity of O20 was 10.21% higher than that of S20. In contrast, the sample with added shortening showed the lowest scavenging activity at each concentration. These results are attributed to the excellent antioxidant properties of orange oil. Limonene, a major component of citrus fruits such as oranges and citrons, has been reported to have excellent antioxidant, anti-inflammatory, antibacterial, and anticancer activities [46,47]. Antioxidants such as ascorbic acid and flavonoids are present in citrus fruits and juices [48], and the chemical composition and antibacterial and antioxidant properties of limonene, a component of orange oil, have been reported [49].

### 3.6. Sensory Test

**Figure 1:**
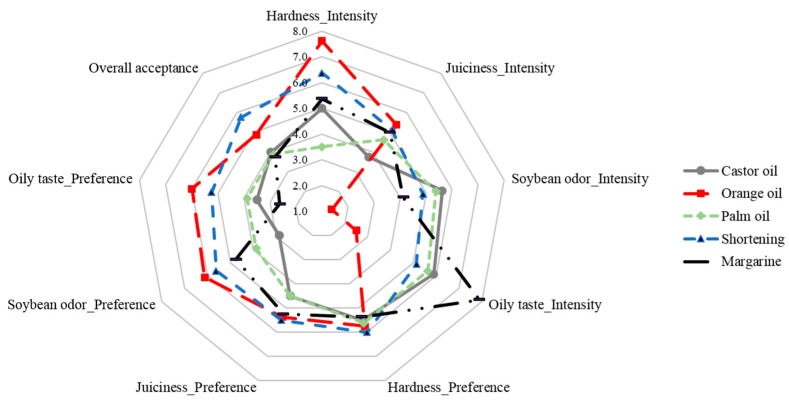
Sensory evaluation of meat analog supplemented with castor oil (●), orange oil (■), palm oil (◆), shortening (▲), and margarine (―).

In this experiment, differences in sensory characteristics between samples of meat analog prepared by adding five types of oil were evaluated. The test results for each sample are shown in Figure 1, along with their intensities and preferences. The sample with orange oil had the lowest soybean odor and oily taste at 1.38 and 2.50 points, respectively, and the highest score for hardness and juiciness at 7.63 and 5.38 points, respectively. In contrast, the castor oil sample had the lowest succulence score (3.75) and soybean odor (5.63). The sample with palm oil had the lowest hardness score (3.50) and the sample with margarine had the highest score (7.88) for oily taste.

The intensity of each sample was reflected in the preference chart. Soybeans are restricted in food development and application because of their peculiar fishy smell [14]. In addition, it is known that succulence is an important factor in judging softness and thus affects palatability [50]. Additionally, in this experiment, in terms of soybean odor and oily taste, the sample with orange oil, with the lowest intensity in the corresponding category, had the highest degree of preference at 6.13 and 6.00 points, respectively; it also had the highest succulence intensity, with a score of 5.38.

Orange oil is a volatile flavor component widely used in the food industry, in beverages, confectionery, desserts, and ice cream [51]. The physicochemical properties of beverage emulsions using the organoleptic properties of orange oil as a flavor component have been evaluated [52]. The addition of volatile flavoring ingredients to improve the fishy smell of pig livers has also been investigated [53]. In this study, the ability of orange oil to mask bean odor and improve juiciness was demonstrated; its application to meat analog is a possibility in the future.

In contrast, although an 11-point scale was used, the highest point for overall acceptance was not high, at 5.75, possibly as no additives such as sugar or salt were added so that the effect of the oil was clear. The overall preference for the sample containing orange oil, evaluated as the highest for succulence, soybean odor, and oily taste, was 4.88 points. In contrast, the overall preference of the sample with shortening was higher, at 5.75 points, possibly due to the influence of other unmeasured sensory characteristics.

## 4. Conclusions

In this study, the physicochemical and sensory characteristics of meat analogs prepared with different vegetable oils were analyzed to find the optimal concentration for improving succulence and soybean odor. The meat analog with orange oil had excellent water content and liquid-holding capacity and showed relatively high hardness; the antioxidant activity was the highest among the oils, and the sensory test showed a reduction in soybean odor, and excellent succulence. Ultimately, to apply this study to industry, specific research will need to establish the optimal concentration of orange oil and the recommended daily intake.

## Figures and Tables

**Table 1:** Ingredients and contents of meat analog.

Sample ^(1)^	Oil Concentration (g/100 g TVP ^(2)^)				
	Castor	Orange	Palm	Shortening	Margarine
C10	10	-	-	-	-
C20	20	-	-	-	-
C30	30	-	-	-	-
C40	40	-	-	-	-
C50	50	-	-	-	-
O10	-	10	-	-	-
O20	-	20	-	-	-
O30	-	30	-	-	-
O40	-	40	-	-	-
O50	-	50	-	-	-
P10	-	-	10	-	-
P20	-	-	20	-	-
P30	-	-	30	-	-
P40	-	-	40	-	-
P50	-	-	50	-	-
S10	-	-	-	10	-
S20	-	-	-	20	-
S30	-	-	-	30	-
S40	-	-	-	40	-
S50	-	-	-	50	-
M10	-	-	-	-	10
M20	-	-	-	-	20
M30	-	-	-	-	30
M40	-	-	-	-	40
M50	-	-	-	-	50

^(1)^ SPI (soy protein isolate) and binder were added (4.5 g and 3 g, respectively, based on 100 g of TVP). ^(2)^ TVP stands for textured vegetable protein composed of Supromax 5050^®^ and Supromax 5010^®^ (1:2).

**Table 2:** Cooking loss (%) of meat analog supplemented with different types or amounts of vegetable oils.

Oil	Concentration (g/100 g TVP)														
	10			20			30			40			50		
Castor oil	10.64	±	0.64 ^Ba^	9.03	±	0.59 ^CDb^	8.92	±	0.20 ^Db^	7.80	±	0.32 ^Dc^	7.55	±	0.45 ^Dc^
Orange oil	9.91	±	0.32 ^Ce^	11.04	±	0.63 ^Ad^	14.76	±	0.64 ^Bc^	16.05	±	0.65 ^Cb^	19.40	±	0.93 ^Ba^
Palm oil	10.26	±	0.62 ^BCb^	8.65	±	0.48 ^Dc^	7.73	±	0.65 ^Ed^	7.34	±	0.50 ^Ed^	17.68	±	0.75 ^Ca^
Shortening	11.63	±	0.64 ^Ad^	9.49	±	0.89 ^BCe^	16.63	±	0.68 ^Ac^	18.01	±	0.64 ^Ab^	20.10	±	1.00 ^Aa^
Margarine	11.26	±	0.84 ^Ad^	9.62	±	0.87 ^Be^	13.95	±	0.78 ^Cc^	16.74	±	0.33 ^Bb^	19.83	±	0.33 ^ABa^

Cooking loss (%) was calculated by the following formula: Cooking loss % = {(W_1_ − W_2_)/W_1_} × 100. W_1_: Weight of sample before cooking (g). W_2_: Weight of sample after cooking (g). Three independent experiments were conducted in this study. ^A–E^ Means within a column with different letters are significantly different (*p* < 0.05). ^a–e^ Means within a row with different letters are significantly different (*p* < 0.05).

**Table 3:** Water contents (%) of meat analog supplemented with different types or amounts of vegetable oils.

Concentration (g/100 g TVP)	Oil	Water Contents (%)						*t*-Value ^(1)^
		Non-Cooking			Cooking			
10	Castor oil	57.52	±	0.39 ^Ca^	57.96	±	0.24 ^Ca^	NS
	Orange oil	61.61	±	0.85 ^Ac^	63.34	±	0.21 ^Ad^	*
	Palm oil	58.78	±	0.58 ^Ba^	58.34	±	0.03 ^Ba^	NS
	Shortening	58.43	±	0.15 ^Ba^	58.39	±	0.12 ^Ba^	NS
	Margarine	58.81	±	0.09 ^Ba^	58.53	±	0.21 ^Ba^	NS
20	Castor oil	52.63	±	0.25 ^Db^	53.61	±	0.07 ^Cb^	**
	Orange oil	64.65	±	0.13 ^Ab^	65.29	±	0.34 ^Ac^	*
	Palm oil	53.51	±	0.28 ^Cb^	52.96	±	0.09 ^Db^	*
	Shortening	54.12	±	0.07 ^Bb^	54.47	±	0.29 ^Bb^	NS
	Margarine	54.40	±	0.08 ^Bb^	54.75	±	0.37 ^Bb^	NS
30	Castor oil	48.91	±	0.48 ^Dc^	49.53	±	0.31 ^Dc^	NS
	Orange oil	65.57	±	0.17 ^Aa^	66.28	±	0.08 ^Ab^	**
	Palm oil	49.41	±	0.49 ^CDc^	50.17	±	0.43 ^Cc^	NS
	Shortening	50.13	±	0.50 ^Cc^	52.59	±	0.11 ^Bc^	**
	Margarine	50.90	±	0.26 ^Bc^	52.34	±	0.26 ^Bc^	**
40	Castor oil	45.79	±	0.29 ^Dd^	45.48	±	0.58 ^Dd^	NS
	Orange oil	66.21	±	0.35 ^Aa^	66.45	±	0.14 ^Ab^	NS
	Palm oil	47.42	±	0.71 ^Cd^	46.68	±	0.44 ^Cd^	NS
	Shortening	47.41	±	0.42 ^Cd^	50.17	±	0.25 ^Bd^	**
	Margarine	48.56	±	0.07 ^Bd^	50.96	±	0.58 ^Bd^	**
50	Castor oil	44.04	±	0.17 ^Ce^	42.68	±	0.25 ^De^	**
	Orange oil	66.29	±	0.50 ^Aa^	67.26	±	0.41 ^Aa^	NS
	Palm oil	45.49	±	0.37 ^Be^	44.32	±	0.12 ^Ce^	**
	Shortening	44.30	±	0.28 ^Ce^	47.14	±	0.46 ^Be^	**
	Margarine	45.82	±	0.41 ^Be^	47.52	±	0.45 ^Be^	**

Water content of sample was calculated by the following formula: Water contents % = {(W_1_ − W_2_)/W_1_} × 100. W_1_: Weight of sample before drying (g). W_2_: Weight of sample after drying (g). Three independent experiments were conducted in this study. ^(1)^ The difference between the non-cooking and cooking samples are presented using the *t*-test. ^A–D^ Means with different superscript letters in the same concentration are significantly different (*p* < 0.05). ^a–e^ Means with different superscript letters in the same oil type are significantly different (*p* < 0.05). NS: Not significant. * *p* < 0.05. ** *p* < 0.01.

**Table 4:** Liquid holding capacity (%) of meat analog supplemented with different types or amounts of vegetable oils stored at 4 °C overnight.

Concentration (g/100 g TVP)	Oil	Liquid Holding Capacity (%)						*t*-Value ^(1)^
		Non-Cooking			Cooking			
10	Castor oil	93.09	±	0.94 ^Ba^	97.70	±	0.85 ^Aa^	**
	Orange oil	96.80	±	0.14 ^Aa^	95.79	±	0.41 ^Ba^	*
	Palm oil	96.53	±	0.32 ^Aa^	96.19	±	0.53 ^Ba^	NS
	Shortening	96.16	±	0.79 ^Aa^	95.98	±	0.73 ^Ba^	NS
	Margarine	96.13	±	0.62 ^Aa^	96.00	±	0.10 ^Ba^	NS
20	Castor oil	92.24	±	0.57 ^Ca^	96.80	±	0.13 ^Aa^	***
	Orange oil	96.11	±	0.03 ^Aab^	94.86	±	0.89 ^Bb^	NS
	Palm oil	96.42	±	0.05 ^Aa^	96.44	±	0.42 ^Aa^	NS
	Shortening	95.52	±	0.20 ^Bab^	96.74	±	0.16 ^Aa^	**
	Margarine	96.51	±	0.03 ^Aa^	96.18	±	0.69 ^Aa^	NS
30	Castor oil	89.38	±	0.61 ^Cb^	95.21	±	0.31 ^Bb^	***
	Orange oil	95.67	±	0.58 ^Ab^	94.20	±	0.08 ^Cbc^	*
	Palm oil	96.27	±	0.74 ^Aa^	96.31	±	0.22 ^Aa^	NS
	Shortening	94.42	±	0.87 ^Bb^	96.28	±	0.58 ^Aa^	*
	Margarine	95.81	±	0.18 ^Aa^	96.45	±	0.17 ^Aa^	*
40	Castor oil	86.86	±	0.63 ^Dc^	92.53	±	0.28 ^Cc^	***
	Orange oil	94.71	±	0.42 ^BCc^	94.01	±	0.30 ^Bbc^	NS
	Palm oil	95.81	±	0.42 ^Aab^	96.41	±	0.29 ^Aa^	NS
	Shortening	95.65	±	0.68 ^ABab^	96.31	±	0.60 ^Aa^	NS
	Margarine	94.29	±	0.51 ^Cb^	94.40	±	0.61 ^Bb^	NS
50	Castor oil	84.19	±	0.29 ^Cd^	86.28	±	0.84 ^Cd^	*
	Orange oil	94.47	±	0.46 ^ABc^	93.84	±	0.45 ^Bc^	NS
	Palm oil	95.21	±	0.74 ^Ab^	96.09	±	0.30 ^Aa^	NS
	Shortening	94.72	±	0.75 ^Ab^	95.89	±	0.34 ^Aa^	NS
	Margarine	93.60	±	0.35 ^Bb^	94.31	±	0.71 ^Bb^	NS

The capacity (%) was calculated by the following formula: Liquid-holding capacity % = {(W_1_ − W_2_)/W_1_} × 100. W_1_: Sample weight before centrifugation (g). W_2_: Sample weight after centrifugation (g). Three independent experiments were conducted in this study. ^(1)^ The difference between the non-cooking and cooking samples are presented using the *t*-test. ^A–D^ Means with different superscript letters in the same concentration are significantly different (*p* < 0.05). ^a–d^ Means with different superscript letters in the same oil type are significantly different (*p* < 0.05). NS: Not significant. * *p* < 0.05. ** *p* < 0.01. *** *p* < 0.001.

**Table 5:** Liquid holding capacity (%) of meat analog supplemented with different types or amounts of vegetable oils stored at 25 °C overnight.

Concentration (g/100 g TVP)	Oil	Liquid Holding Capacity (%)						*t*-Value ^(1)^
		Non-Cooking			Cooking			
10	Castor oil	92.67	±	0.63 ^Ba^	92.85	±	0.43 ^Ca^	NS
	Orange oil	94.54	±	0.17 ^Aa^	94.31	±	0.46 ^Ba^	NS
	Palm oil	93.12	±	0.07 ^Ba^	95.25	±	0.30 ^Aa^	**
	Shortening	91.34	±	0.97 ^Ca^	94.45	±	0.12 ^Ba^	*
	Margarine	93.48	±	0.79 ^ABa^	93.90	±	0.23 ^Ba^	NS
20	Castor oil	91.89	±	0.61 ^BCa^	92.66	±	0.87 ^Ba^	NS
	Orange oil	94.40	±	0.10 ^Aa^	94.07	±	0.53 ^Aa^	NS
	Palm oil	92.92	±	0.57 ^Ba^	94.44	±	0.25 ^Aab^	*
	Shortening	90.41	±	0.95 ^Da^	90.03	±	0.20 ^Cb^	NS
	Margarine	90.97	±	0.68 ^CDb^	90.41	±	0.09 ^Cb^	NS
30	Castor oil	91.50	±	0.82 ^Ba^	91.33	±	0.59 ^Bb^	NS
	Orange oil	93.40	±	0.86 ^Ab^	93.71	±	0.09 ^Aab^	NS
	Palm oil	91.74	±	0.81 ^Bb^	94.15	±	0.18 ^Ab^	*
	Shortening	88.64	±	0.37 ^Cb^	88.93	±	0.78 ^Cc^	NS
	Margarine	88.35	±	0.42 ^Cc^	87.38	±	0.29 ^Cc^	*
40	Castor oil	82.61	±	0.22 ^Eb^	89.91	±	0.50 ^Bc^	***
	Orange oil	92.81	±	0.29 ^Ab^	93.26	±	0.05 ^Ab^	NS
	Palm oil	90.77	±	0.43 ^Bc^	92.60	±	0.66 ^Ac^	*
	Shortening	86.93	±	0.59 ^Cc^	84.91	±	0.15 ^Cd^	**
	Margarine	85.77	±	0.85 ^Dd^	84.48	±	0.12 ^Cd^	NS
50	Castor oil	77.24	±	0.80 ^Dc^	85.18	±	0.45 ^Cd^	***
	Orange oil	92.89	±	0.36 ^Ab^	93.28	±	0.18 ^Ab^	NS
	Palm oil	81.91	±	0.30 ^Cd^	88.43	±	0.94 ^Bd^	***
	Shortening	85.33	±	0.09 ^Bd^	82.90	±	0.75 ^De^	**
	Margarine	82.35	±	0.19 ^Ce^	80.92	±	0.09 ^Ee^	***

The capacity (%) was calculated by the following formula: Liquid-holding capacity % = {(W_1_ − W_2_)/W_1_} × 100. W_1_: Sample weight before centrifugation (g). W_2_: Sample weight after centrifugation (g). Three independent experiments were conducted in this study. ^(1)^ The difference between the non-cooking and cooking samples are presented using the *t*-test. ^A–E^ Means with different superscript letters in the same concentration are significantly different (*p* < 0.05). ^a–e^ Means with different superscript letters in the same oil type are significantly different (*p* < 0.05). NS: Not significant. * *p* < 0.05. ** *p* < 0.01. *** *p* < 0.001.

**Table 6:** Liquid holding capacity (%) of meat analog supplemented with different types or amounts of vegetable oils stored at 35 °C overnight.

Concentration (g/100 g TVP)	Oil	Liquid Holding Capacity (%)						*t*-Value ^(1)^
		Non-Cooking			Cooking			
10	Castor oil	90.91	±	0.88 ^Ba^	94.26	±	0.61 ^Aa^	**
	Orange oil	92.46	±	0.25 ^Aa^	92.33	±	0.19 ^Ca^	NS
	Palm oil	92.91	±	0.74 ^Aa^	93.22	±	0.45 ^Ba^	NS
	Shortening	87.85	±	0.28 ^Ca^	92.25	±	0.54 ^Ca^	***
	Margarine	91.28	±	0.14 ^Ba^	90.58	±	0.35 ^Da^	*
20	Castor oil	87.98	±	0.70 ^Bb^	93.85	±	0.27 ^Aa^	***
	Orange oil	91.14	±	0.43 ^Ab^	90.60	±	2.03 ^Bab^	NS
	Palm oil	91.52	±	0.54 ^Ab^	92.18	±	0.51 ^ABb^	NS
	Shortening	84.57	±	0.30 ^Cb^	88.24	±	0.22 ^Cb^	***
	Margarine	88.25	±	0.15 ^Bb^	87.38	±	0.33 ^Cb^	*
30	Castor oil	86.70	±	0.22 ^Bc^	91.96	±	0.16 ^Ab^	***
	Orange oil	90.16	±	0.24 ^Ac^	89.97	±	0.34 ^Bb^	NS
	Palm oil	90.74	±	0.97 ^Ab^	91.55	±	0.55 ^Ab^	NS
	Shortening	83.45	±	0.67 ^Dc^	84.73	±	0.87 ^Cc^	NS
	Margarine	84.52	±	0.27 ^Cc^	85.34	±	0.12 ^Cc^	**
40	Castor oil	85.65	±	0.38 ^Bd^	91.78	±	0.77 ^Ab^	***
	Orange oil	88.83	±	0.59 ^Ad^	88.83	±	0.49 ^Bbc^	NS
	Palm oil	88.37	±	0.75 ^Ac^	88.76	±	0.30 ^Bc^	NS
	Shortening	82.09	±	0.58 ^Cd^	80.07	±	0.59 ^Dd^	*
	Margarine	78.02	±	0.37 ^Dd^	81.55	±	0.34 ^Cd^	***
50	Castor oil	76.23	±	0.42 ^Ce^	86.32	±	0.20 ^Bc^	***
	Orange oil	88.62	±	0.28 ^Ad^	88.14	±	0.31 ^Ac^	NS
	Palm oil	80.33	±	0.67 ^Bd^	87.70	±	0.33 ^Ad^	***
	Shortening	80.82	±	0.44 ^Be^	77.12	±	0.36 ^Ce^	***
	Margarine	75.23	±	0.40 ^De^	77.22	±	0.23 ^Ce^	**

The capacity (%) was calculated by the following formula: Liquid-holding capacity % = {(W_1_ − W_2_)/W_1_} × 100. W_1_: Sample weight before centrifugation (g). W_2_: Sample weight after centrifugation (g). Three independent experiments were conducted in this study. ^(1)^ The difference between the non-cooking and cooking samples are presented using the *t*-test. ^A–D^ Means with different superscript letters in the same concentration are significantly different (*p* < 0.05). ^a–e^ Means with different superscript letters in the same oil type are significantly different (*p* < 0.05). NS: Not significant. * *p* < 0.05. ** *p* < 0.01. *** *p* < 0.001.

**Table 7:** Texture profile analysis (hardness, adhesiveness, and cohesiveness) of meat analog supplemented with different types or amounts of vegetable oils.

	Oil	Concentration (g/100 g TVP)														
		10			20			30			40			50		
Hardness (N)	Castor oil	39.82	±	1.02 ^Aa^	32.53	±	1.81 ^Bb^	29.95	±	1.33 ^Ac^	23.59	±	0.38 ^Ad^	15.71	±	0.27 ^Be^
	Orange oil	41.44	±	1.53 ^Aa^	36.84	±	1.06 ^Ab^	29.36	±	0.61 ^Ac^	25.49	±	1.09 ^Ad^	23.05	±	0.60 ^Ae^
	Palm oil	36.16	±	3.74 ^Ba^	27.37	±	0.91 ^Cb^	26.10	±	0.73 ^Bb^	16.75	±	1.69 ^Cc^	16.46	±	2.00 ^Bc^
	Shortening	31.27	±	3.25 ^Ca^	24.57	±	1.20 ^Db^	22.58	±	2.23 ^Cb^	21.24	±	2.65 ^Bb^	12.41	±	1.87 ^Cc^
	Margarine	29.35	±	0.89 ^Ca^	26.36	±	0.55 ^Cb^	19.82	±	0.57 ^Dc^	17.54	±	0.49 ^Cd^	14.85	±	0.42 ^Be^
Adhesiveness (mJ)	Castor oil	0.03	±	0.05 ^Ab^	0.10	±	0.00 ^Ab^	0.15	±	0.17 ^Ab^	0.18	±	0.10 ^Ab^	0.40	±	0.16 ^Aa^
	Orange oil	0.10	±	0.08 ^Aa^	0.05	±	0.06 ^Aa^	0.15	±	0.24 ^Aa^	0.00	±	0.00 ^Ca^	0.05	±	0.10 ^Ba^
	Palm oil	0.05	±	0.06 ^Ab^	0.03	±	0.05 ^Ab^	0.15	±	0.10 ^Aa^	0.00	±	0.00 ^Cb^	0.03	±	0.05 ^Bb^
	Shortening	0.05	±	0.06 ^Aa^	0.08	±	0.15 ^Aa^	0.03	±	0.05 ^Aa^	0.05	±	0.06 ^BCa^	0.15	±	0.06 ^Ba^
	Margarine	0.03	±	0.05 ^Ab^	0.05	±	0.06 ^Ab^	0.10	±	0.00 ^Aab^	0.10	±	0.00 ^ABab^	0.18	±	0.10 ^Ba^
Cohesiveness	Castor oil	0.33	±	0.04 ^Aa^	0.34	±	0.06 ^Aba^	0.32	±	0.03 ^BCa^	0.22	±	0.02 ^Cb^	0.23	±	0.01 ^Bb^
	Orange oil	0.29	±	0.01 ^Bc^	0.31	±	0.03 ^Bbc^	0.29	±	0.02 ^Cc^	0.33	±	0.03 ^Aab^	0.36	±	0.01 ^Aa^
	Palm oil	0.31	±	0.04 ^ABab^	0.35	±	0.02 ^ABa^	0.33	±	0.01 ^Bab^	0.30	±	0.01 ^Bb^	0.34	±	0.03 ^Aa^
	Shortening	0.33	±	0.01 ^Ac^	0.38	±	0.02 ^Ab^	0.43	±	0.03 ^Aa^	0.36	±	0.01 ^Abc^	0.34	±	0.02 ^Ac^
	Margarine	0.30	±	0.01 ^ABc^	0.35	±	0.02 ^ABb^	0.42	±	0.02 ^Aa^	0.35	±	0.03 ^Ab^	0.34	±	0.01 ^Ab^

Texture profile analysis was conducted using the cubed sample with a width, length, and height of 2 cm after standing to cool. Ten independent experiments were conducted in this study. ^A–D^ Means within a column with different letters are significantly different (*p* < 0.05). ^a–e^ Means within a row with different letters are significantly different (*p* < 0.05).

**Table 8:** Texture profile analysis (springiness, gumminess, and chewiness) of meat analog supplemented with different types or amounts of vegetable oils.

	Oil	Concentration (g/100 g TVP)														
		10			20			30			40			50		
Springiness (mm)	Castor oil	7.25	±	0.55 ^Aa^	6.80	±	0.58 ^Aab^	6.53	±	0.31 ^Ab^	4.50	±	0.35 ^Cc^	4.11	±	0.37 ^Dc^
	Orange oil	6.81	±	0.31 ^Ab^	6.50	±	0.90 ^Ab^	6.64	±	0.40 ^Ab^	6.84	±	0.19 ^Ab^	7.66	±	0.26 ^Aa^
	Palm oil	6.81	±	0.62 ^Aab^	7.39	±	0.44 ^Aa^	7.02	±	0.22 ^Aa^	6.31	±	0.28 ^Bb^	6.32	±	0.32 ^Bb^
	Shortening	6.98	±	0.36 ^Aab^	7.19	±	0.35 ^Aa^	6.56	±	0.39 ^Abc^	6.29	±	0.23 ^Bc^	5.63	±	0.35 ^Cd^
	Margarine	6.66	±	0.24 ^Aab^	7.08	±	0.42 ^Aa^	6.95	±	0.34 ^Aa^	6.19	±	0.43 ^Bbc^	5.66	±	0.40 ^Cc^
Gumminess (N)	Castor oil	13.37	±	2.35 ^Aa^	11.12	±	2.49 ^Aab^	9.49	±	0.65 ^ABb^	3.43	±	0.28 ^Cc^	2.38	±	0.56 ^Dc^
	Orange oil	11.82	±	1.10 ^ABa^	11.24	±	1.22 ^Aa^	8.07	±	1.15 ^ABb^	8.54	±	1.74 ^Ab^	10.87	±	0.74 ^Aa^
	Palm oil	11.21	±	1.25 ^ABCa^	9.50	±	0.42 ^ABab^	8.50	±	1.86 ^ABb^	4.91	±	0.58 ^Bc^	5.57	±	1.05 ^Bc^
	Shortening	10.40	±	1.14 ^BCa^	8.74	±	1.20 ^Bab^	10.01	±	1.68 ^Aa^	7.59	±	0.92 ^Ab^	4.26	±	0.45 ^Cc^
	Margarine	8.99	±	0.65 ^Ca^	8.31	±	0.39 ^Bab^	7.72	±	0.37 ^Bb^	6.12	±	0.36 ^Bc^	5.14	±	0.46 ^BCd^
Chewiness (mJ)	Castor oil	97.9	±	23.1 ^Aa^	76.8	±	22.8 ^Aab^	62.1	±	6.0 ^Ab^	15.5	±	2.4 ^Dc^	9.9	±	3.0 ^Dc^
	Orange oil	80.8	±	10.8 ^ABa^	73.9	±	17.0 ^Aab^	53.3	±	4.4 ^Ac^	58.3	±	11.0 ^Abc^	83.3	±	6.0 ^Aa^
	Palm oil	76.7	±	14.0 ^ABCa^	70.3	±	6.4 ^Aab^	59.8	±	13.5 ^Ab^	31.0	±	3.9 ^Cc^	35.4	±	8.5 ^Bc^
	Shortening	72.4	±	5.5 ^BCa^	63.0	±	9.9 ^Aa^	65.7	±	11.9 ^Aa^	48.0	±	7.5 ^ABb^	24.1	±	3.6 ^Cc^
	Margarine	56.6	±	5.1 ^Ca^	59.4	±	3.8 ^Aa^	57.1	±	2.6 ^Aa^	41.1	±	8.2 ^BCb^	29.2	±	4.2 ^BCc^

Texture profile analysis was conducted using the cubed sample with a width, length, and height of 2 cm after standing to cool. Ten independent experiments were conducted in this study. ^A–D^ Means within a column with different letters are significantly different (*p* < 0.05). ^a–d^ Means within a row with different letters are significantly different (*p* < 0.05).

**Table 9:** pH values of vegetable oils used to prepare meat analog.

Oil	pH		
Castor oil	5.64	±	0.02 ^D^
Orange oil	3.19	±	0.04 ^E^
Palm oil	5.88	±	0.03 ^C^
Shortening	6.31	±	0.04 ^B^
Margarine	6.51	±	0.05 ^A^

Three independent experiments were conducted in this study. ^A–E^ Means within a column with different letters are significantly different (*p* < 0.05).

**Table 10:** DPPH radical scavenging activity (%) of meat analog supplemented with different types or amounts of vegetable oils.

Oil	Concentration (g/100 g TVP)														
	10			20			30			40			50		
Castor oil	22.12	±	0.72 ^Cd^	24.27	±	2.40 ^Ccd^	26.22	±	0.59 ^Cbc^	27.66	±	0.42 ^Cab^	29.22	±	0.75 ^Ca^
Orange oil	30.15	±	0.41 ^Ae^	32.54	±	1.01 ^Ad^	33.96	±	0.35 ^Ac^	35.36	±	0.57 ^Ab^	36.91	±	0.28 ^Aa^
Palm oil	28.41	±	1.08 ^Bd^	29.33	±	0.84 ^Bcd^	30.19	±	0.52 ^Bbc^	31.46	±	0.37 ^Bb^	33.46	±	1.21 ^Ba^
Shortening	20.07	±	1.11 ^Dd^	22.33	±	0.42 ^Cc^	25.21	±	1.84 ^Cb^	27.57	±	0.34 ^Ca^	28.13	±	0.47 ^Ca^
Margarine	21.67	±	0.80 ^Cd^	23.01	±	0.60 ^Cc^	26.21	±	0.51 ^Cb^	27.91	±	0.46 ^Ca^	28.65	±	0.94 ^Ca^

The activity was calculated by the following formula: DPPH radical scavenging activity % = [1 − {(A1 − A2)/A3}] × 100. A1: Absorbance of 0.1 mL DPPH solution + 0.1 mL sample solution. A2: Absorbance of 0.1 mL methanol + 0.1 mL sample solution. A3: Absorbance of 0.1 mL DPPH + 0.1 mL methanol. Three independent experiments were conducted in this study. ^A–D^ Means within a column with different letters are significantly different (*p* < 0.05). ^a–e^ Means within a row with different letters are significantly different (*p* < 0.05).

## Data Availability

Data is contained within the article.
